# Search for a correlation between telomere length and severity of retinitis pigmentosa due to the dominant rhodopsin Pro23His mutation

**Published:** 2009-03-27

**Authors:** Dyonne T. Hartong, Terri L. McGee, Michael A. Sandberg, Eliot L. Berson, Folkert W. Asselbergs, Pim van der Harst, Immaculata De Vivo, Thaddeus P. Dryja

**Affiliations:** 1 Ocular Molecular Genetics Institute, Harvard Medical School, Massachusetts Eye and Ear Infirmary, Boston, MA; 2 The Berman-Gund Laboratory for the Study of Retinal Degenerations, Harvard Medical School, Massachusetts Eye and Ear Infirmary, Boston, MA; 3 Department of Ophthalmology, University Medical Center Groningen, University of Groningen, Groningen, The Netherlands; 4 Department of Cardiology, University Medical Center Groningen, University of Groningen, Groningen, The Netherlands; 5 Channing Laboratory, Department of Medicine, Brigham and Women's Hospital, Harvard Medical School, Boston, MA

## Abstract

**Purpose:**

Great variation exists in the age of onset of symptoms and the severity of disease at a given age in patients with retinitis pigmentosa (RP). The final pathway for this disease may involve apoptotic photoreceptor cell death. Telomere length is associated with biologic aging, senescence, and apoptosis. We evaluated whether the length of telomeres in leukocytes correlated with the severity of RP in patients with the Pro23His rhodopsin mutation who have shown marked heterogeneity in disease severity.

**Methods:**

We evaluated 122 patients with the Pro23His rhodopsin mutation. The patients’ retinal function was stratified according to their 30-Hz cone electroretinogram (ERG). The length of telomeres in leukocytes was measured by the quantitative real time polymerase chain reaction (qRT–PCR) method in the 15 patients with the highest age-adjusted 30-Hz ERG amplitudes and in the 15 patients with the lowest amplitudes.

**Results:**

Mean leukocyte telomere length was similar in the 15 patients with the highest cone ERG amplitudes (median: 0.40 units; interquartile range 0.36–0.56) and the 15 patients with the lowest cone amplitudes (median: 0.41 units; inter quartile range 0.34 −0.64; p=0.95).

**Conclusions:**

We found no evidence for an association between telomere length and the severity of RP as monitored by the cone ERG in patients with the Pro23His rhodopsin mutation.

## Introduction

Retinitis pigmentosa (RP) is a group of inherited retinal degenerations with progressive photoreceptor cell death typically causing night blindness, constricted visual fields, and in later stages, a decrease in visual acuity. As the condition progresses, the cone electroretinogram (ERG) amplitude decreases. Mutations in the rhodopsin gene (*RHO*; OMIM ID: +180380) account for about 25% of the dominantly inherited RP cases and less than a few percent of recessively inherited cases [1-4]. The Pro23His mutation is the most frequently reported rhodopsin mutation in the United States [5], accounting for about 8.5% of all dominant RP cases or about 1/3 of those with a dominant rhodopsin mutation [6]. Interfamilial and intrafamilial variation in disease severity among patients with this mutation have been described [7,8], suggesting that factors besides the primary gene defect contribute to the disease.

Telomeres are structures at the ends of chromosome arms consisting of tandem repeats of the nucleotide sequence TTAGGG. These repetitive elements stabilize chromosomes by preventing fusion with other chromosome ends and by impeding degradation of coding DNA [9-11]. Short telomere length has been associated with apoptosis [12-14]. Telomere length is dependent on the number of previous cell divisions and, thus, decreases with age. This decrease is compensated in part by telomerase, which adds TTAGGG tandem repeats to the 3′ end of the DNA strand [15-19]. Telomere length is highly variable among individuals, and this variation is detectable at birth [20-22]. Telomere lengths are similar in different tissues of the same individual, so the analysis of one cell type (e.g., leukocytes) reflects the telomere size throughout that individual [23,24].

Previous studies have shown an inverse relationship between leukocyte telomere length and the occurrence of age-related diseases such as chronic heart failure [25] and dementia [26]. Shorter telomere length has also been associated with disease severity [27].

In this study we evaluated the possible association between telomere length and the severity of RP. We hypothesized that individuals with shorter telomere lengths may have more severe photoreceptor degeneration. We evaluated 122 patients who had autosomal dominant RP due to the Pro23His mutation and selected 15 patients with the best preserved retinal function and the 15 who had the least preserved retinal function. Telomere length was compared with the loss of retinal function as indicated by the amplitude of the 30-Hz cone ERG.

## Methods

### Patient selection

This study conformed to the Declaration of Helsinki and was approved by the Institutional Review Boards of Harvard Medical School and the Massachusetts Eye and Ear Infirmary and the Human Subjects Committee of the Harvard School of Public Health. Informed consent was obtained from all patients. We evaluated 122 RP patients previously found to have the dominant mutation Pro23His in *RHO* [28]. Patients were recruited from the files of the Berman-Gund Laboratory, Harvard Medical School. Patients had elevated final dark adaptation thresholds and reduced rod ERGs; all had attenuated retinal arterioles and most had intraretinal bone spicule pigment around the periphery. The general health of the patients was good. Blood samples from the patients were obtained through phlebotomy and leucocyte DNA was isolated using a Phenol/Chloroform extraction.

### Measurements of retinal function

Retinal function was evaluated based on the ERG amplitude recorded in response to 30-Hz white flashes. Full-field ERG responses were obtained following pupil dilation using one drop of a solution containing 10% phenylephrine hydrochloride and 1% cyclopentolate hydrochloride (Akorn, inc., Buffalo Grove, IL) and 45 min of dark adaptation. ERGs were recorded with a contact lens electrode placed on the cornea topically anesthetized with one drop of 0.5% proparacaine hydrochloride (Alcon Laboratories, Inc. Fort Worth, TX). Responses below 10 μV were recorded with narrow band-pass filtering and then computer averaged to increase the signal to noise ratio as described previously [29,30]. Amplitudes were averaged between the two eyes (when results from both eyes were available) and adjusted for age and refractive error.

### Telomere lengths

The relative telomere lengths were determined using a modified quantitative real time polymerase chain reaction (qRT–PCR) [31,32]. Briefly, the 7900 HT thermocycler (Applied Biosystems, Foster city, CA) was used to obtain the relative length of telomeres, expressed as the ratio between the repeat copy number of telomeres (T) and a reference single-copy gene (S; 36B4 gene, chromosome 12). All samples were compared with the same reference DNA sample. This method has been shown to correlate with Southern blot measurements of telomere length [33,34].

For each reaction 5 ng of DNA were dried in the well of a reaction plate and resuspended in 10 μl of PCR reaction mix, which contained 1X Qiagen Quantitect Sybr Green Master Mix, 2.5 mM of dithiothreitol, and primers. Primers for the telomere reaction were 270 nM of the sense primer (GGT TTT TGA GGG TGA GGG TGA GGG TGA GGG TGA GGG T) and 900 nM of the antisense primer (TCC CGA CTA TCC CTA TCC CTA TCC CTA TCC CTA TCC CTA). Primers for the 36B4 reaction were 300 nM of the sense primer (CAG CAA GTG GGA AGG TGT AAT CC) and 500 nM of the antisense primer (CCC ATT CTA TCA TCA ACG GGT ACA A). The temperature for the first 5 min was 95 °C; this was followed by 40 cycles consisting of 15 s at 95 °C and 2 min at 54 °C for the telomere reaction or 1 min at 58 °C for the 36B4 reaction. ABI 7900HT software calculated the cycle threshold for the Telomere (T) and reference gene (S) for each sample. The ratio T:S represented the relative amount of telomere DNA compared to single copy DNA and therefore corresponded to relative telomere length. All samples were analyzed in triplicate and the coefficient of variation (CV), describing the amount of repeat variability, was calculated.

### Statistical analysis

We confined our analysis of telomere length to sets of patients at the extremes of disease severity as determined by cone 30-Hz ERG amplitudes. Individuals at the extremes for a continuously variable trait (such as disease severity) provided the most power for uncovering the responsible factors, since they are most likely to differ in the level of the responsible factors or their frequency (see, for example, power calculations for mapping genes for quantitative traits [35]). First, the relative telomere lengths (T:S ratios) in the high and low ERG amplitude groups were compared by means of a two-tailed Mann–Whitney U test. Second, a linear regression analysis compared the log_e_ 30-Hz ERG amplitude to the T:S ratio adjusting for age and refractive error. The Spearman correlation was used to test the association between age and telomere length. A p-value of <0.05 was considered statistically significant. The aforedescribed calculations were performed with SPSS version 14 software (SPSS, Chicago, IL) or with JMP, version 6 (SAS Institute, Cary, NC).

## Results

### Patients

We ranked 122 patients with the *RHO*-Pro23His mutation according to their mean 30-Hz ERG amplitude, adjusted for age and refractive error. After excluding a few outlier patients with respect to age, we selected the 15 with the highest 30-Hz ERG amplitude and the 15 with lowest. The 15 patients with the least severe RP (i.e., those with the highest ERG amplitudes) consisted of nine males and six females. The 15 patients with the most severe disease (i.e., those with the lowest ERG amplitudes) comprised eight males and seven females. The mean age at time of phlebotomy for DNA samples from these two groups was 46.7 years (range: 27–58) and 45.1 years (range: 31–62), respectively. Many of the 122 patients were related to others in this set. Of the 30 individuals included in the analysis of the least and most severely affected, 14 were first degree relatives (siblings). An additional nine individuals were also related to others in the analysis set but were more distantly related. Among the first degree relatives, four sets of two siblings appeared in the group with high ERG amplitudes (least severe), one set of three siblings appeared in the group with low ERG amplitudes (most severe), and one sibship was split among the groups with two siblings in the most severe group and one in the least severe group. Seven patients were not related to any of the 30 extreme patients in the in the analysis set. No unaffected controls were included since we were only interested in the variation of telomere length related to disease severity within this group of Pro23His mutants. For patient characteristics and individual results, see Table 1.

**Table 1:** Patients included in high and low ERG amplitude groups.

**Sample ID**	**Family ID**	**1° relative**	**Sex**	**Age (years)**	**Av30Hz ERG (μV)**	**T/S ratio**
**High ERG amplitude group**						
218–288	5938	218–289	f	34	81	0.430198
001–191	1566	-	m	38	76.0*	0.537613
218–289	5938	218–288	m	41	69.5	0.557743
226–1070	6149	226–1063	m	49	67.5	0.364527
226–1063	6149	226- 1070	m	51	51.5	0.350156
226–007	5850	218–005, 218–002	f	56	50	0.756707
218–309	6281	-	f	46	46	0.376173
226–685	5970	001–299	m	56	42.5	0.397979
001–299	5970	226–285	m	58	41.1	0.341629
226–638	6149	-	f	27	41	0.380276
218–243	6038	226–905	m	52	38	0.394993
226–905	6038	218–243	f	50	38	0.556106
001–385	6803	-	m	46	35.3	0.519532
218–282	5938	-	m	47	27.5	0.56872
218–280	E716	-	f	49	12.6	0.311241
**Low ERG amplitude group**						
001–007	6994	-	f	34	2.3	0.364527
226–1426	5998	-	f	42	1.04	0.769996
001–162	6653	-	f	46	0.7	0.368992
218–003	5850	-	m	38	0.65	0.412048
001–390	1509	-	m	48	0.47	0.376173
218–005	5850	226–007, 218–002	m	55	0.3	0.608039
001–131	6281	-	m	43	0.3	0.355099
218–255	5938	001–387, 218–031	f	32	0.23	0.586807
001–089	6149	-	f	31	0.2*	0.799895
218–407	6888	-	f	62	0.17	0.274106
218–060	1566	-	f	62	0.16	0.340283
001–078	6038	-	m	49	0.15	0.197481
001–387	5938	218–255, 218–031	m	34	0.13*	0.651994
218–002	5850	226–007, 218–005	m	60	0.12	0.430762
218–031	5938	218–255, 001–387	m	41	0.08	0.638473

This table lists the 30 Hz cone ERG amplitudes averaged across both eyes (Av30Hz ERG) for the 15 patients included in the high ERG amplitude group (least severely affected) and the 15 patients included in the low ERG amplitude group (most severely affected) along with the relative telomere lengths (ie. T/S ratio) obtained for each patient, the patient’s age at phlebotomy, their gender and any first degree relatives also included in this study. Age ranges, gender distribution and T/S ratios were similar between the two groups whereas the 30 Hz ERG amplitudes were on average nearly 100 fold greater in the high ERG group than the low ERG group. Amplitudes marked with an * indicate that data was available for only one eye. All patients had reduced or nondetectable rod ERG amplitudes (data not shown); some still had normal cone ERG amplitudes (normal range=50–125 μV).

### Analysis of the relative telomere lengths

**Figure 1:**
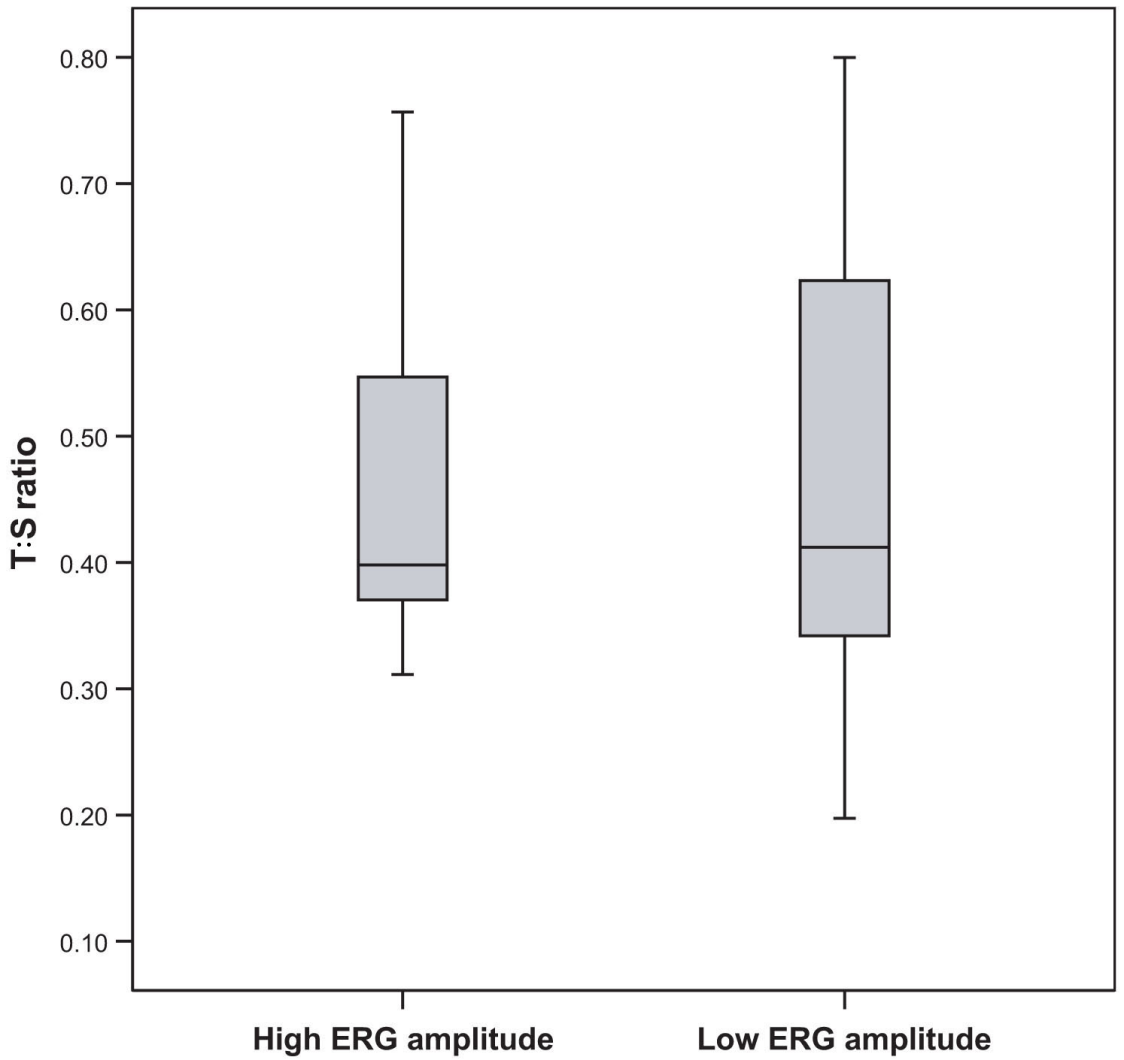
Relative telomere lengths in Pro23His RP patients. Boxplot showing no difference in T:S ratio’s between patients with high- or low ERG amplitudes. The shaded boxes indicate the interquartile range. The horizontal line in each shaded box denotes the median, and the error bars mark the upper and lower 95 percentiles of the T:S ratio.

The coefficient of variation in our study was satisfactorily low with 1.27% for the T assay and 0.64% for the S assay. Spearman’s correlation test showed a modest inverse relationship between patient age and relative telomere length in the 30 patients (r=-0.38; p=0.037). We found no significant difference in T:S ratio between the 15 patients with the highest cone ERG amplitudes (median: 0.40 units; interquartile range 0.36–0.56) and the 15 patients with the lowest cone ERG amplitudes (median: 0.41 units; interquartile range 0.34 −0.64; p=0.95) using the Mann–Whitney U nonparametric test (Figure 1). The results did not change when first degree relatives were excluded from the analysis (n=22): T:S ratios of 11 patients with highest cone ERG amplitudes (median: 0.39 units; interquartile range 0.36–0.56) were similar to the ratios of 11 patients with the lowest cone ERG amplitudes (median: 0.36 units; interquartile range 0.33 −0.64; p=0.38). Multiple regression analysis of the total group of 30 patients, adjusting for age and refractive error, also showed no significant relation between log_e_ 30 Hz-ERG amplitude and telomere T:S ratio (t=-0.75; p=0.46).

## Discussion

The rhodopsin gene product is a transmembrane G-coupled protein (opsin). It is found in the rod outer segments, and, when bound with chromophore, mediates the initial steps of the phototransduction cascade [36,37]. The *RHO*-Pro23His mutation encodes a misfolded protein that aggregates within the endoplasmic reticulum [38,39] and seems to activate apoptosis by the unfolded protein response (UPR) [40-42]. On average, patients with the *RHO*-Pro23His mutation tend to have milder disease compared to those with other rhodopsin mutations [43-47]. However, there is great variability in disease severity among those with Pro23His, and this variation can be objectively measured with ERGs [48].

Since photoreceptors in RP appear to die ultimately through apoptosis, and since cells with chromosomes with short telomeres are prone to apoptosis, we hypothesized that patients with short telomeres might have more severe disease because their photoreceptors would more rapidly undergo apoptosis in response to the deleterious effects of *RHO*-Pro23His. To test this hypothesis, we confined our analysis to sets of patients at the extremes of disease severity as determined by 30-Hz cone ERG amplitudes. We found no evidence for an association between telomere length and severity of RP. However, a limitation of our analysis method must be noted: We used DNA derived from dividing leukocytes, since our cells of interest, the nondividing retinal photoreceptors, were not available from living patients. Although it is reported that telomere size is highly correlated among tissues [49,50], it is known that dividing cells are subject to changes in telomere length with the main known factor being age. Since our groups of patients with mild and severe RP were of about the same ages, and since the effect of age is relatively small compared to the individual differences in telomere length, we assumed that the telomere length in the peripheral leucocytes reflected the telomere length in nondividing photoreceptor cells. However, a possible difference in telomere lengths between these two cell types cannot be ruled out. Our method using qRT–PCR measurements of telomere lengths has successfully been used in other studies and has been shown to correlate to the Southern blot method of telomere measurement [51,52]. However both methods provide only an estimate of actual telomere length.
